# Supplementary material for: Determination of Conformational and Functional Stability of Potential Plague Vaccine Candidate in Formulation
Source: Vaccines (Basel). 2022 Dec 23;11(1):27. doi: 10.3390/vaccines11010027 (PMC9865242; doi:10.3390/vaccines11010027)
Supplement: Supplementary file 1 [file vaccines-11-00027-s001.zip › vaccines-2044844-supplementary.pdf]

Table S1 Second derivative Amide I peak assignments on the FTIR spectrum of LcrV formulation (PD1)

Designation	Approximate Frequency (cm^{-1})	Description
Extended structure	1610	Stronger intermolecular hydrogen bonds
3_{10} helices	1663	Intermediary conformation of sorts, and provides insight into the initiation of α-helix folding
α-helix	1659	Hydrogen bonding between hydrogen in amino acid and carboxyl group leading to folding
β-sheets	1627	Produces the strongest inter-strand stability as it allows the inter-strand hydrogen bonds between carbonyls and amines

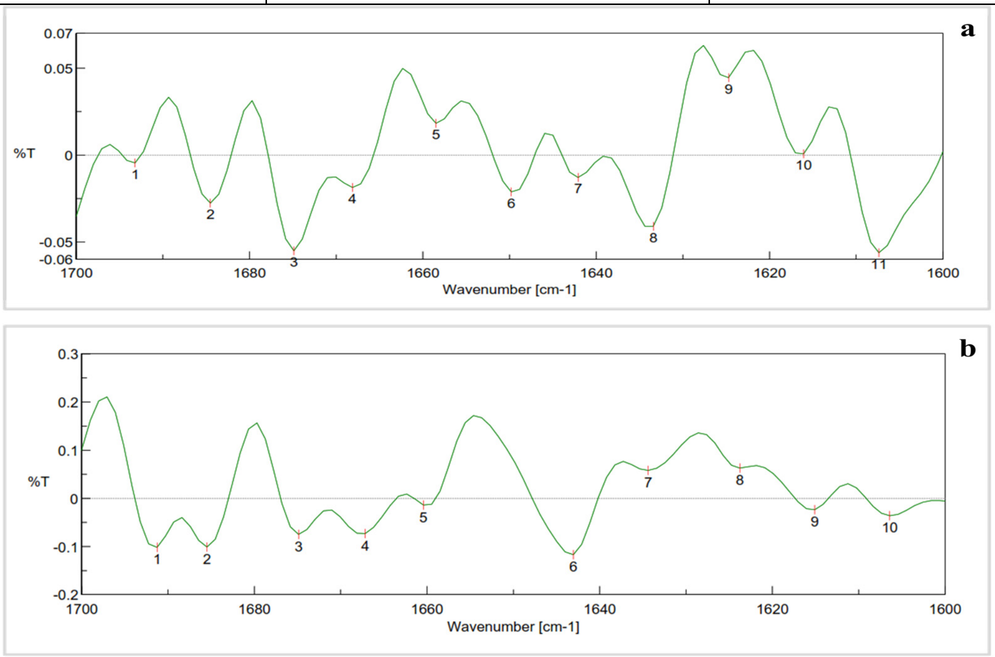

Figure S1: Second Derivative Amide I (C=O Stretching) Spectrum of a) LcrV and b) PD1 formulation after three weeksof exposure to room temperature, showing 3_{10} helices (at 1663 cm^{-1}), α helix (at 1659 cm^{-1}), and β sheets (at 1627 cm^{-1})

a

[Result of Peak Picking]

No.	Position	Intensity	No.	Position	Intensity	No.	Position	Intensity
1	3957.21	97.4719	2	3935.04	92.0616	3	3910.93	91.2118
4	3887.79	91.8421	5	3856.93	87.8946	6	3790.4	82.8154
7	3747.98	87.0829	8	3718.08	90.5952	9	3676.62	94.1873
10	3606.23	75.4896	11	3343	15.085	12	3308.29	15.1672
13	3261.04	15.4887	14	2904.27	88.5299	15	2835.81	95.9021
16	2809.78	96.9462	17	2765.42	98.6819	18	2691.18	101.039
19	2615	101.095	20	2592.82	99.3858	21	2536.9	99.1331
22	2513.76	98.2629	23	2444.33	98.8163	24	2386.48	80.3811
25	2361.41	4.89622	26	2341.16	52.4718	27	2297.77	76.0181
28	2263.06	83.5497	29	2204.24	75.6983	30	2162.78	79.0971
31	2109.74	85.0133	32	2061.53	94.2819	33	2018.14	70.849

[Result of Peak Picking]

No.	Position	Intensity	No.	Position	Intensity	No.	Position	Intensity
34	1973.79	73.9392	35	1908.22	96.2461	36	1893.75	95.783
37	1858.08	94.4867	38	1817.58	95.7948	39	1758.76	94.0803
40	1726.94	96.4923	41	1624.73	88.0025	42	1561.09	98.797
43	1529.27	96.1484	44	1482.03	91.3319	45	1439.6	91.519
46	1409.71	92.2526	47	1381.75	92.2786	48	1358.6	93.389
49	1233.25	91.8386	50	1179.26	91.3052	51	1120.44	87.6482
52	1080.91	87.261	53	962.305	92.2395	54	916.986	88.0173
55	889.023	83.9215	56	869.739	84.1064	57	832.133	78.8353
58	814.777	79.3582	59	783.922	73.172	60	757.888	72.7752
61	739.567	70.1976	62	714.497	70.5055	63	684.606	67.3676
64	657.607	75.5367	65	630.609	67.1718	66	588.182	66.1542
67	564.077	71.1788	68	551.542	70.5791	69	525.507	29.7742
70	504.294	23.4059	71	474.403	50.1318	72	440.655	37.8011
73	410.763	27.5343						

b

[Result of Peak Picking]

No.	Position	Intensity	No.	Position	Intensity	No.	Position	Intensity
1	3958.18	96.3858	2	3932.14	93.4782	3	3885.86	92.6304
4	3855.97	93.01	5	3829.94	92.8916	6	3786.54	89.7563
7	3743.15	92.7283	8	3729.66	91.9648	9	3718.08	91.111
10	3701.69	93.2139	11	3663.12	88.9143	12	3630.34	94.0327
13	3606.23	90.0079	14	3340.1	22.7612	15	2986.23	70.5098
16	2900.41	80.3477	17	2830.99	97.5577	18	2734.57	101.895
19	2693.1	102.121	20	2648.75	102.446	21	2588	101.392
22	2538.83	100.009	23	2510.86	99.4274	24	2442.4	95.0901
25	2367.19	52.836	26	2354.66	36.7047	27	2323.8	78.4368
28	2309.34	89.4936	29	2295.84	89.7184	30	2262.09	88.4289
31	2219.67	86.5687	32	2202.31	85.6	33	2181.1	87.8124

[Result of Peak Picking]

No.	Position	Intensity	No.	Position	Intensity	No.	Position	Intensity
34	2159.88	77.216	35	2141.56	86.47	36	2111.67	80.2736
37	2066.35	85.9268	38	2045.14	81.0979	39	2022	87.2291
40	2011.39	86.7932	41	1997.89	86.6472	42	1972.82	87.9093
43	1955.47	89.4388	44	1938.11	88.4682	45	1894.72	92.5802
46	1873.51	93.6696	47	1797.33	95.7095	48	1765.51	96.9713
49	1631.48	65.3967	50	1529.27	95.791	51	1481.06	94.0611
52	1437.67	93.0602	53	1391.39	87.1019	54	1252.54	91.1735
55	1065.48	77.1829	56	967.126	93.9725	57	915.058	93.3182
58	893.844	91.461	59	867.81	94.0354	60	833.098	91.9458
61	787.779	90.7314	62	758.852	91.6836	63	739.567	92.251
64	711.604	96.9901	65	690.391	95.1817	66	654.715	96.6072
67	630.609	89.0378	68	593.004	86.7949	69	579.504	91.1131
70	564.077	85.9568	71	550.577	75.6795	72	524.543	28.9698
73	502.366	23.2408	74	472.474	28.8514	75	457.047	18.8213
76	441.619	21.6325	77	420.406	20.2199			

c

[Result of Peak Picking]

No.	Position	Intensity	No.	Position	Intensity	No.	Position	Intensity
1	3959.14	99.6476	2	3934.07	98.0342	3	3885.86	97.7342
4	3850.18	98.8996	5	3806.79	94.6198	6	3701.69	84.6654
7	3355.53	12.7065	8	2988.16	62.7573	9	2902.34	81.1923
10	2838.7	99.2694	11	2692.14	103.105	12	2646.82	103.43
13	2592.82	101.51	14	2427.94	97.8382	15	2401.91	98.834
16	2353.69	31.1509	17	2321.87	90.0306	18	2282.34	87.4759
19	2261.13	84.9797	20	2221.59	90.4082	21	2188.81	92.4959
22	2160.85	81.2369	23	2143.49	91.7798	24	2111.67	89.8932
25	2069.25	91.226	26	2042.25	91.758	27	2019.1	91.7661
28	1999.82	93.8854	29	1972.82	94.0576	30	1937.15	93.3885
31	1896.65	93.3779	32	1860.97	92.9493	33	1832.04	93.5711

[Result of Peak Picking]

No.	Position	Intensity	No.	Position	Intensity	No.	Position	Intensity
34	1795.4	94.5896	35	1764.55	94.511	36	1725.01	89.9984
37	1630.52	62.4336	38	1531.2	94.7841	39	1481.06	93.6934
40	1439.6	94.2429	41	1406.82	92.1884	42	1383.68	92.5483
43	1250.61	95.2813	44	1184.08	97.6347	45	1076.08	90.2767
46	967.126	99.9842	47	892.88	96.635	48	832.133	93.6522
49	787.779	88.4664	50	758.852	89.6412	51	729.925	87.1066
52	687.498	83.9574	53	656.643	90.2771	54	620.002	89.7843
55	591.075	91.0009	56	565.041	97.0707	57	549.613	92.824
58	522.615	35.6488	59	501.401	20.1329	60	473.439	22.8821
61	455.118	19.9266	62	441.619	18.0157	63	422.334	15.5067

Figure S2: Second Derivative Amide II (C-N Stretching) Data corresponding to peaks between 1600-1500 $cm^{-1}$of a) LcrV b) Polymer Dextran and c) PD1 formulation

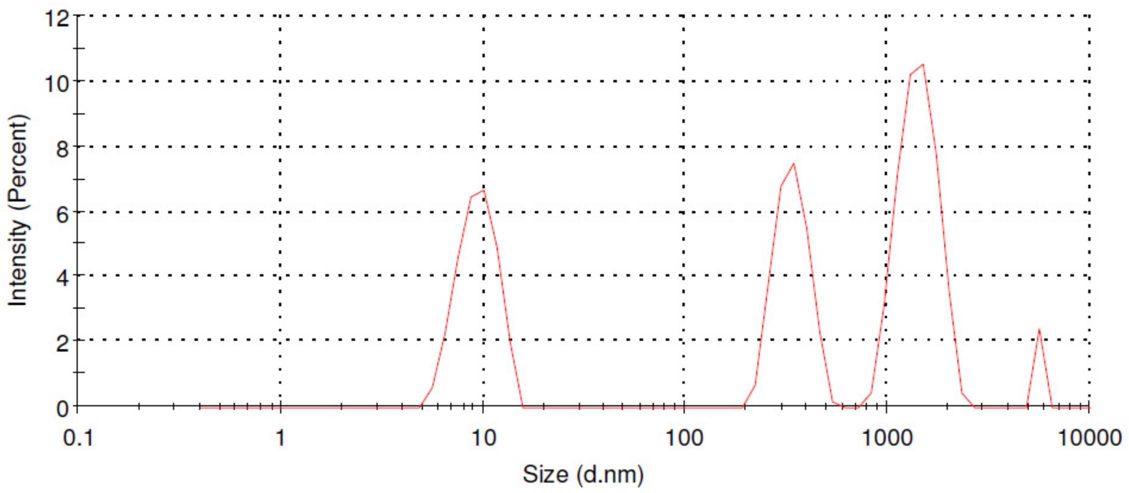

Figure S3: Particle size analysis of LcrV+NaCl Formulationafter eight days of exposure to room temperature

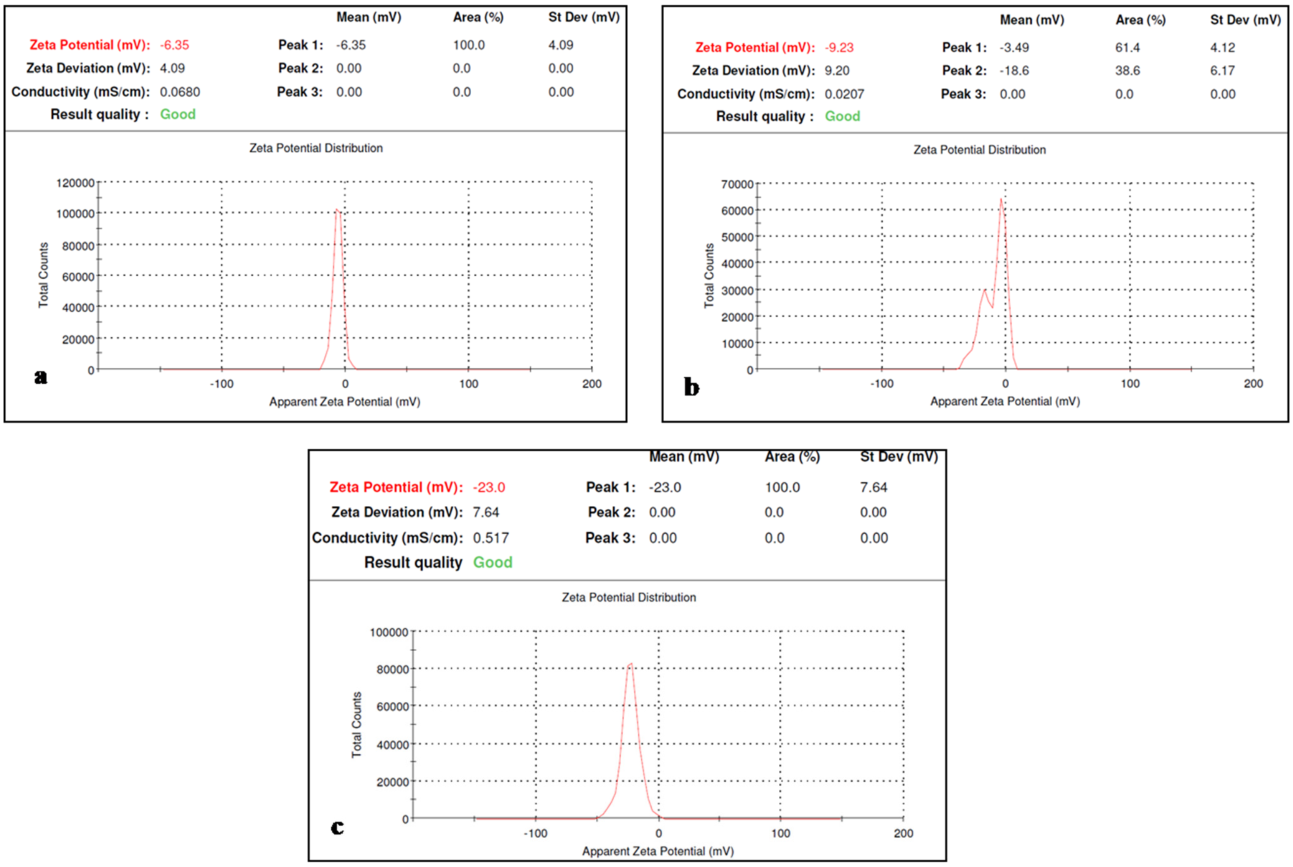

Figure S4: Zeta Potential analysis of a) LcrV b) Dextran and c) PD1 Formulation after three weeks of exposure to room temperature

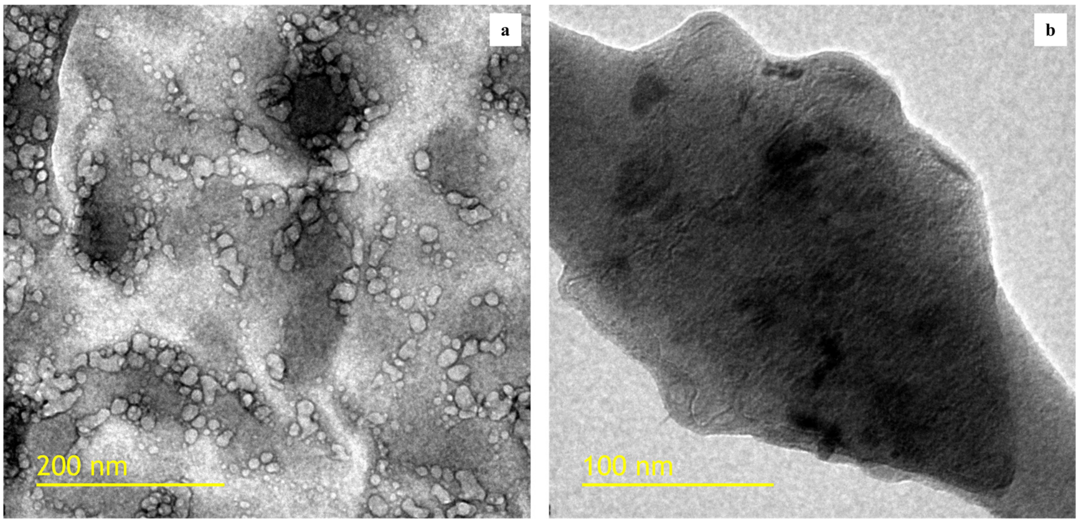

Figure S5: TEM analysis of a) LcrV and b) PD1 Formulation after three weeks of exposure to room temperature.
